# Interleukin-26 expression in tuberculosis disease and its regulatory effect in macrophage polarization and intracellular elimination of *Mycobacterium tuberculosis*

**DOI:** 10.3389/fcimb.2024.1455819

**Published:** 2024-10-04

**Authors:** Kaisong Huang, Haijin Zhou, Mei Chen, Rui Chen, Xiaoping Wang, Qi Chen, Zhiyun Shi, Yanfang Liang, Luxin Yu, Ping Ouyang, Li Li, Dan Jiang, Guangxian Xu

**Affiliations:** ^1^ Guangdong Provincial Key Laboratory of Medical Immunology and Molecular Diagnostics, School of Medical Technology, Guangdong Medical University, Dongguan, China; ^2^ Dongguan Key Laboratory of Molecular Immunology and Cell Therapy, Guangdong Medical University, Dongguan, China; ^3^ Reference Lab, Fourth People’s Hospital of Ningxia Hui Autonomous Region, Yinchuan, China; ^4^ School of Life Sciences, Ningxia University, Yinchuan, China; ^5^ Department of Pathology, Dongguan Binhaiwan Central Hospital, Dongguan, China

**Keywords:** Tuberculosis, interleukin-26, M1 macrophages, ROS, *Mycobacterium tuberculosis*

## Abstract

Tuberculosis(TB), an infectious disease caused by *Mycobacterium tuberculosis* (Mtb) infections, remains the leading cause of mortality from a single infectious agent globally. The progression of tuberculosis disease is contingent upon the complex interplay between the host’s immune system and the pathogen Mtb. Interleukin-26 (IL-26), the most recently identified cytokine belonging to the IL-10 family, exhibits both extracellular antimicrobial properties and pro-inflammatory functions. However, the precise role of IL-26 in the host immune defense against Mtb infections and intracellular killing remains largely unexplored. In this study, we observed significantly elevated IL-26 mRNA expression in peripheral blood mononuclear cells of active-TB patients compared to healthy individuals. Conversely, circulating IL-26 levels in the plasma of adult TB patients were markedly lower than those of healthy cohorts. We purified recombinant IL-26 from an *E*. coli expression system using the Ni-NTA resin. Upon stimulations with the recombinant IL-26, human THP1 cells exhibited rapid morphological changes characterized by increased irregular spindle shape and formation of granular structures. Treating THP1 cells with IL-26 can also lead to heightened expressions of *CD80*, *TNF-α*, and *iNOS* but not *CD206* and *Arg1* in these cells, indicating an M1 macrophage differentiation phenotype. Furthermore, our investigations revealed a dose-dependent escalation of reactive oxygen species production, decreased mitochondrial membrane potential, and enhanced autophagy flux activity in THP1 macrophages following IL-26 treatment. Moreover, our results demonstrated that IL-26 contributed to the elimination of intracellular Mycobacterium tuberculosis via orchestrated ROS production. In conclusion, our findings elucidated the role of IL-26 in the development of tuberculosis and its contributions to intracellular bacilli killing by macrophages through the induction of M1-polarization and ROS production. These insights may have significant implications for understanding the pathogenesis of tuberculosis and developing novel therapeutic strategies.

## Introduction

Tuberculosis(TB), a chronic infectious disease caused by *Mycobacterium tuberculosis*(Mtb), is the leading cause of death from a single infectious agent, accounting for over one million deaths annually worldwide (Chakaya et al., 2021). It is estimated that approximately 2 billion individuals globally have been chronically infected with Mtb, with 5 to 10 percent of them progressing to active tuberculosis during their lifetime, thereby posing a significant global public health challenge (Houben and Dodd, 2016). The Bacillus Calmette-Guérin (BCG), the solely licensed and wildly inoculated vaccine worldwide, has shown only moderate protective effects against severe extrapulmonary forms of tuberculosis in young children, like infant meningitis, but proved to be ineffective against pulmonary tuberculosis in adolescents and adults (Griffiths et al., 2016; Mangtani et al., 2014). The treatment of drug-susceptible Mtb typically requires a regimen of four drugs taken for six months. However, the emergence and widespread prevalence of drug-resistant Mtb strains and HIV co-infection have compromised the efficacy of existing TB medications, further complicating the current challenging situation (Seung et al., 2015). The interplay between the host immune system and Mtb virulence factors plays a pivotal role in determining the infection outcomes. Therefore, a comprehensive understanding of their intricate interactions may generate novel insights into the development of more effective TB vaccines and therapies.

Both the innate and adaptive immune systems are involved in the control of Mycobacterium tuberculosis infections. Cytokines released from innate immune cells and functional CD4 subset cells play a crucial role in regulating and activating the functions of these immune cells to eliminate Mtb infections. Interleukin-26(IL-26), a novel member of the IL-10 family, which also includes IL-10, IL-19, IL-22, and IL-24, was first discovered in human T lymphocytes infected with hybridizing Herpesvirus saimiri (HVS) *in vitro* (Knappe et al., 2000). In humans, the *IL-26* gene is situated on chromosome 12q15, between the interferon-gamma (*IFN-γ*) and *IL-22* gene. The 171 amino acids of humans IL-26 can form six highly cationic α-helices in structure, with the capacity to form dimers and higher-order multimers, which is highly conserved across the mammalian species (Knappe et al., 2000; Tengvall et al., 2016). Although primarily secreted by Th17 cells, IL-26 production has also been documented in other immune cell types, including Th1 cells, CD8^+^ cells, NK cells, and macrophages (Corridoni et al., 2020; Dambacher et al., 2009; Fujii et al., 2017). The IL-26 receptor comprises two subunits, namely the IL-20 receptor α- subunit (IL-20RA) and IL-10 receptor β- subunit (IL-10RB), with intracellular signaling likely conveyed through the STAT1, STAT3, ERK, JNK, and AKT signaling pathways (Dambacher et al., 2009; Hor et al., 2004; Lin et al., 2020; Sheikh et al., 2004; Tengvall et al., 2016). Currently, IL-26 is believed to be involved in a variety of chronic inflammatory and autoimmune disorders diseases, including Crohn’s disease, rheumatoid arthritis, atopic dermatitis, and asthma, as elevated levels of IL-26 have been commonly observed in the development of these diseases (Corvaisier et al., 2012; Fries et al., 2023; Fujii et al., 2017). Furthermore, due to its distinctive amino acid composition and structure, which are characterized by the clustering of cationic charges and the formation of multimers, IL-26 also exhibits properties akin to cationic antimicrobial peptides like the human cathelicidin LL-37 (Hansen et al., 2021; Larochette et al., 2019). *In-vitro* studies have shown that IL-26 can bind to the cell envelopes of germs and lead to the formation of pores and disruptions of cell membranes (Meller et al., 2015; Rivas-Santiago et al., 2013; Zasloff, 2002). Nonetheless, the precise role of IL-26 in the pathogenesis of tuberculosis disease remains largely unknown.

It is well established that macrophages, phagocytic innate immune cells responsible for bacterial elimination and acting as the bridge between innate and adaptive immune response, serve as the primary niche for the survival and replication of intracellular Mycobacterium tuberculosis in the lung tissues of infected individuals (Sable et al., 2019; Stamm et al., 2019). Nevertheless, the impact of IL-26 on the activation and intracellular pathogens killing capacity of macrophages, particularly its role in eradicating invading Mycobacterium tuberculosis and the potential associated mechanisms, remains elusive. Therefore, given the multifunctional effects of IL-26 on chronic disease progression and the knowledge gap of IL-26 in macrophage activations and functionality, this study aimed to explore the clinical relevance and implications of IL-26 in tuberculosis disease progressions, as well as to elucidate the effect of IL-26 on the activation and killing ability of human macrophage for the invading Mtb bacilli, along with uncovering the underlying molecular mechanisms involved.

## Materials and methods

### Cell culture, bacterial culture and infections

The human THP-1 cell line was acquired from the ATCC Cell Bank of the Chinese Academy of Sciences (Shanghai, China). The cell lines were cultured at 37°C with 5% CO_2_ in RPMI 1640 medium (BI, Israel) supplemented with 10% fetal bovine serum (BI, Israel). To differentiate THP1 monocytes into macrophages, the THP-1 cells were simulated with 80 ng/ml PMA for 48h and allowed to rest for 24h prior to other experiments. *Mycobacterium tuberculosis* H37Ra strain was routinely cultured either in Middlebrook 7H9 broth supplemented with 0.2% glycerol, 0.05% tween-80, and 10% OADC with shaking at 100 rpm or on Middlebrook 7H10 plates supplemented with 0.5% glycerol and 10% OADC. *Escherichia coli* (*E*. coli) DH5α strain, used for gene cloning, and BL21 (DE3) strain, used for protein expression, were grown in Luria-Bertani (LB) liquid medium with shaking at 200 rpm or plated on LB agar. The THP1 cells were infected with *M*. tuberculosis H37Ra at a multiplicity of 10:1 for 6 hours, and the supernatants or cells were collected at indicated time points for subsequent assays, as detailed below.

### Reagents and antibodies

The pET-28a vector was acquired from Addgene. Restriction endonucleases NcoI, XhoI, and T4 ligase were obtained from Takara (Beijing, China). The Agarose Gel DNA Purification kit and plasmid extraction kit were purchased from Tiangen (Beijing, China). Isopropyl-β-D-thiogalactoside (IPTG), N-acetylcysteine (NAC), the JC-1 probe of mitochondrial membrane potential assay kit, the Anti-His-tag antibody, and the DCFH-DA probe were obtained from Beyotime (Shanghai, China). The MitoTracker Red Fluorescent Probe CMXRos was purchased from BioLib (Beijing, China). The Ni-NTA column was sourced from Genscript (Nanjing, China), while *E*. coli BL21 (DE3) was obtained from Vazyme (Nanjing, China). Phorbol-12-myristate 13-acetate(PMA), chloroquine (CQ), and rapamycin were purchased from Sigma-Aldrich (Shanghai, China). The Trizol reagent, RevertAid First Strand cDNA Synthesis Kit, and SYBR Green PCR kit were sourced from Thermo Fisher (USA). The anti-CD80-PE antibody, anti-CD206-FITC antibody, DifcoTM Middlebrook 7H9 Agar, and OADC enrichment solution were procured from BD (USA). TNF-α and IL-10 ELISA kits were obtained from CUSABIO (Wuhan, China). The anti-β-actin, anti-LC3, HRP-conjugated anti-rabbit lgG or anti-mice lgG secondary antibodies were sourced from CST (Wuhan, China). The anti-IL-26 monoclonal antibody was obtained from Abcam (UK). The secondary antibodies of FITC-conjugated anti-mice-lgG and Cy3-conjugated anti-rabbit-lgG were obtained from Beyotime (Shanghai, China). The mitochondrial MitoSOX probe was obtained from Meilun (Dalian, China), and DAPI(4’,6-Diamidino-2-Phenylindole) was purchased from Solarbio (Beijing, China).

### Patients and clinical samples

IL-26 was primarily produced and co-expressed with IL-22 by Th1, Th17, and NK cells of the PBMC populations. In this study, we collected twenty-five cases of peripheral blood samples from active pulmonary tuberculosis patients, alongside 29 cases of peripheral blood samples from healthy individuals undergoing routine physical examination at The Fourth People’s Hospital of Ningxia Hui Autonomous Region from 2019 to 2020. The blood plasma was isolated by centrifuging at 2,000 rpm for 10 min and stored in 1 ml aliquots at -80°C until analysis. Peripheral blood mononuclear cells (PBMC) were separated using a Ficoll density gradient, as previously reported (Zhang et al., 2016). Lung tissue samples were obtained via surgical resection from individuals with severe tuberculosis-related pulmonary complications at Dongguan Binhaiwan Central Hospital. Subsequently, the resected lung tissues were sectioned into small pieces for Histopathological analysis using Hematoxylin and Eosin (HE) staining and for fluorescent microscopy analysis after staining with the anti-IL-26 monoclonal antibody and the Cy3-conjugated secondary antibody. Written informed consent has been obtained from all participants, and the research ethics committee of the respective institution approved this study.

### Cloning, expression, and purification of recombinant IL-26 protein

The commercially available IL-26 protein is produced using the E. coli system. In our study, we retrieved the original sequence encoding the mature peptide of the human *IL-26* gene from the GenBank database (accession number NM_018402.2). This sequence was then codon-optimized to match the codon usage preference of *Escherichia coli* to enhance heterologous expression. Subsequently, the codon-optimized sequence was commercially synthesized and inserted into the pET-28a expression vector following digestion with NcoI and XhoI restriction enzyme. The resulting expression plasmid was then transformed into *E*. coli BL21 (DE3) strain. Expression of recombinant IL-26 was induced at 37°C for six hours with the addition of 0.4 mM isopropyl-beta-d-thiogalactopyranoside (IPTG). The overexpressed recombinant IL-26 protein was purified using immobilized metal affinity chromatography with nickel resin. The purified protein was dialyzed in phosphate-buffered saline (PBS) overnight at 4°C. The expression and purification of IL-26 were conducted by ZoonBio Biotechnology (Nanjing, China) under our delegation. The purity of the purified IL-26 was assessed using sodium dodecyl sulfate-polyacrylamide gel electrophoresis (SDS-PAGE). Additionally, the recombinant IL-26 was further validated by Western blotting with both anti-His and anti-IL-26 monoclonal antibodies.

### Cell viability assays

Macrophage viability was assessed using the Cell Counting kit-8 (CCK-8) as previously reported (Saito et al., 2006). Briefly, the THP-1 cell (1.5×10^4^) was firstly seeded into a 96-well plate and treated with 80 ng/ml PMA for 48h at 37°C in a humidified atmosphere with 5% CO_2_. Subsequently, the THP-1 macrophages were stimulated with different concentrations of IL-26 (0-30 μg/ml) for 12h, 24h, and 48h, respectively. Following a 2-hour incubation with CCK-8 solution, the absorbance of cells at 450nm was quantified using a Microplate Reader. The percentage of cell viability was normalized to that of unstimulated cells.

### Flow cytometry

THP-1 macrophages (5×10^4^) stimulated with different concentrations (0-2μg/mL) of IL-26 were harvested at indicated time points, and then these cells were stained by PE-conjugated Anti-CD80 and FITC conjugated Anti-CD206 antibodies. Subsequently, the cells were analyzed using an LSR II flow cytometer equipped with FACSDiva software (BD Bioscience). Acquired data were processed using the FlowJo software.

### ELISA and qRT-PCR assays

The purified recombinant IL-26 was obtained as described above. To assess cytokine expressions triggered by IL-26, THP-1 cells were seeded into 12-well plates at a density of 5 × 10^5^ cells per well. Following differentiations by PMA, the cells were washed and treated with varying concentrations (0-2μg/mL) of IL-26. Cell culture supernatants and cell lysates were collected at 6, 12, and 24 hours post-stimulation. Total RNA from the above cell lysate or PBMC cells isolated from clinical blood samples were extracted using the Trizol reagent. After removing potential DNA contamination by DNase I, the cDNA was synthesized using random primers from the reverse transcription kit (Thermo Fisher, China). Transcript levels of different genes were quantified using the SYBR Green kit (Qiagen, China) on an ABI7500 real-time instrument. The GAPDH (glyceraldehyde-3-phosphate dehydrogenase) gene served as an internal control, and the relative gene expressions of CD80, TNF-α, iNOS, CD206, Arg-1, IL-10, and IL-26 (PBMC cells) were determined using the 2^−ΔΔCt^ method. The specific primers used are listed in **Supplementary Table 1**. The concentrations of secreted TNF-α and IL-10 in the supernatants of stimulated THP1 cells, as well as the IL-26 in the plasma of active tuberculosis patients or healthy control individuals, were quantified using commercially available Enzyme-linked immunosorbent assay (ELISA) kit (Abcam, China) following the manufacturer’s instructions.

### Mitochondrial activity and membrane potential assay

The mitochondrial activity and membrane potential of THP1 cells were assessed using the MitoTracker probe and the JC-1 membrane potential kit following the manufacturer’s instructions (Beyotime, China). Briefly, THP-1 cells (1.5×10^4^) were seeded into 96-well cell culture plates in RPMI 1640 medium supplemented with 10% FBS and differentiated by 80ng/ml PMA as described above. Following 24 hours of resting, the THP1 macrophages were simulated with varying concentrations of IL-26 for 24 hours. Subsequently, the cells were stained with MitoTracker Red Fluorescent Probe CMXRos or JC-1 probe, a lipophilic cationic dye. Subsequently, the fluorescence was examined using both a BioTek Synergy HT plate reader and fluorescence microscopy.

### Determination of intracellular ROS and mitochondrial ROS production

To quantify cells’ ROS levels, THP1 macrophages, pre-incubated with or without 20 mM NAC for 2 hours, were stimulated with varying concentrations of IL-26, as described earlier. At the indicated time points, cells were harvested and stained with 5 uM of the fluorescent probe DCFH-DA or 1.25μM MitoSOX Red at 37°C for 20 minutes in an HBSS buffer, respectively. Subsequently, cell fluorescence intensity was assessed using flow cytometry or measured by a fluorescent microplate reader.

### SDS-PAGE and western blotting analysis

Human THP-1 macrophages were lysed in RIPA lysis buffer containing protease inhibitor PMSF. Protein concentrations were determined using the Pierce BCA protein assay kit (Beyotime, China) to ensure uniform loading. The equal lysate was then separated on 12% SDS-PAGE gels and transferred to a polyvinylidene difluoride membrane. Following blocking with 5% BSA for one hour at room temperature, the membrane-bound protein was incubated with an anti-LC3-A/B antibody overnight at 4°C. After washing the membrane three times with TBST buffer, the membrane-bound protein was further probed with the corresponding secondary antibody for one hour. Protein bands were visualized using enhanced chemiluminescence (ECL) reagents (Thermo Fisher, China) and captured on a chemiluminescence detection system(Bio-Rad China).

### Confocal fluorescence microscopy analysis

For protein immunofluorescence staining, 1×10^5^ human THP-1 cells were seeded on glass coverslips in 24-well plates and stimulated by IL-26 as described above. Following fixation with 4% paraformaldehyde for 15 min, the cells were permeabilized with 0.2% TritionX-100 in PBS for 10 min and then blocked with 10% BSA in PBS for one hour at room temperature. Subsequently, THP1 macrophages were incubated with the probes of either DCFH-DA(5uM) or MitoSOX Red (1.25uM), Mitrotracker, JC-1, or with the primary antibody of anti-LC3b (1:100 dilution, Mouse mAb) for overnight at 4°C. For LC3 staining analysis, the cells were further incubated with FITC-conjugated goat anti-mouse IgG antibody after three times PBS washing. Nuclei were stained with DAPI for 10 min at room temperature. The coverslips were mounted on glass slides using a fluorescence mounting solution, and stained cells were examined and imaged using laser confocal microscopy (Zeiss, Germany).

### CFU assays

PMA-differentiated THP-1 macrophages were pre-treated with varying concentrations of IL-26 for 24 hours, and then THP1 cells were infected with Mycobacterium tuberculosis H37Ra at a multiplicity of 10 for 6 hours at 37°C. Subsequently, extracellular bacteria were removed by washing three times with pre-warmed PBS. At 24 hours post-infection, cells were washed and then lysed in PBS containing 0.2% Triton X-100 for 20 min. Serial 10-fold dilutions of cell lysates were plated on 7H10 agar containing 10% OADC and incubated for three weeks at 37°C for bacterial enumeration. To combine the treatment of THP-1 cells with 2µg/ml IL-26 with either 20 mM N-acetylcysteine (NAC) or 25 µM Chloroquine (CQ), NAC and CQ were added 2 hours prior to the Mtb infections.

### Statistical analysis

Experiments were performed in triplicate, and the data shown are representative of at least three independent experiments, expressed as mean ± standard deviation (SD). The unpaired, two-tailed Student’s t-test, or one-way ANOVA with Tukey’s multiple comparison testing, was used to assess the statistical significance of experiment groups using GraphPad Prism 8 software. The differences were considered statistically significant when the P-value was less than 0.05.

## Results

### Levels of IL-26 in plasma and IL-26 mRNA expression in PBMCs of active tuberculosis patients

Elevated expressions of IL-26 have been documented in a series of chronic inflammatory and autoimmune disorders, including Crohn’s disease and rheumatoid arthritis. However, its expression levels and correlations with active tuberculosis remain largely unexplored. To bridge this knowledge gap, we quantified IL-26 levels in plasma and IL-26 mRNA expressions in PBMCs from 25 patients diagnosed with active pulmonary tuberculosis and 29 healthy controls using the ELISA and RT-PCR methods, respectively. All these 29 healthy individuals were younger than 60 years old. Of the 25 tuberculosis patients, four are older than 60. The demographic information for these recruited TB patients and healthy controls was presented as **Supplementary Data Sheet 1**.

**Figure 1:**
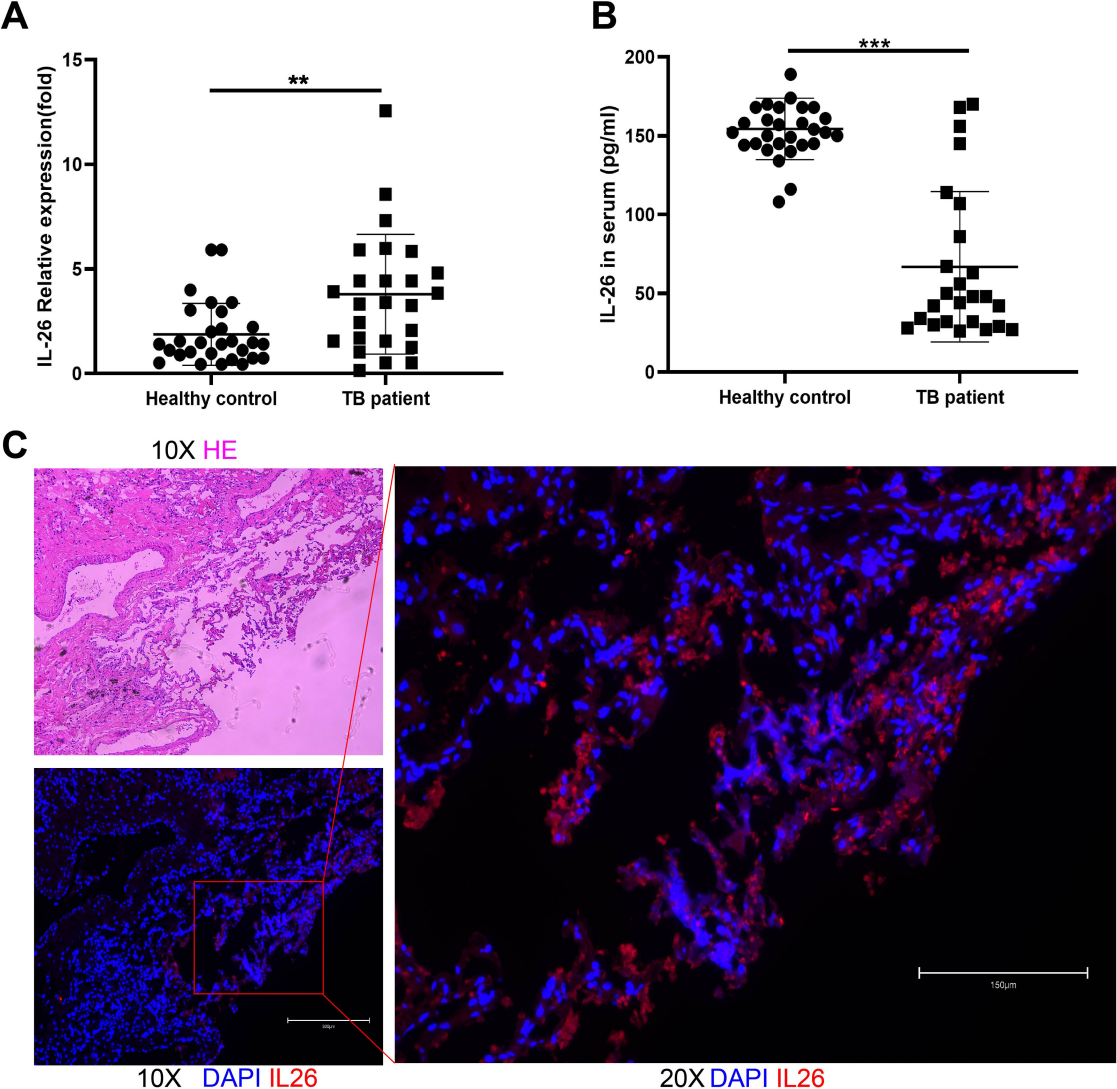
IL-26 expression levels in individuals with active pulmonary tuberculosis and healthy cohorts. **(A)** IL-26 mRNA expression in PBMCs from TB patients was significantly higher than that of healthy individuals. **(B)** Circulating plasma- IL-26 level in TB patients was substantially lower than that of healthy control. **(C)** HE and Immunofluorescence staining of lung tissue sections from TB patients. The IL-26 protein was stained with a monoclonal anti-IL-26 antibody and further by a Cy3-conjugated secondary antibody (red). The nuclei were stained with DAPI (blue). **p < 0.01, ***p < 0.001 (Student’s t-test).

Interestingly, our findings demonstrated significantly elevated IL-26 mRNA expression in PBMCs from pulmonary TB patients compared to healthy individuals (**Figure 1A**). Conversely, plasma levels of IL-26 were reduced in TB patients in comparison to those in the healthy control cohort (**Figure 1B**). The expressions of IL-26 in lung granuloma tissue of individuals with active TB patients were also examined using the immunostaining method. After hematoxylin and eosin staining, typical granuloma structures were identified in these tissue sections from TB patients (**Figure 1C**). Additionally, we also observed an abundant presence of IL-26 (red) at the periphery of large granulomas in lung tissue from tuberculosis patients (**Figure 1C**).

### Expression, purification, and cytotoxic effect of recombinant IL-26 on macrophage viability

**Figure 2:**
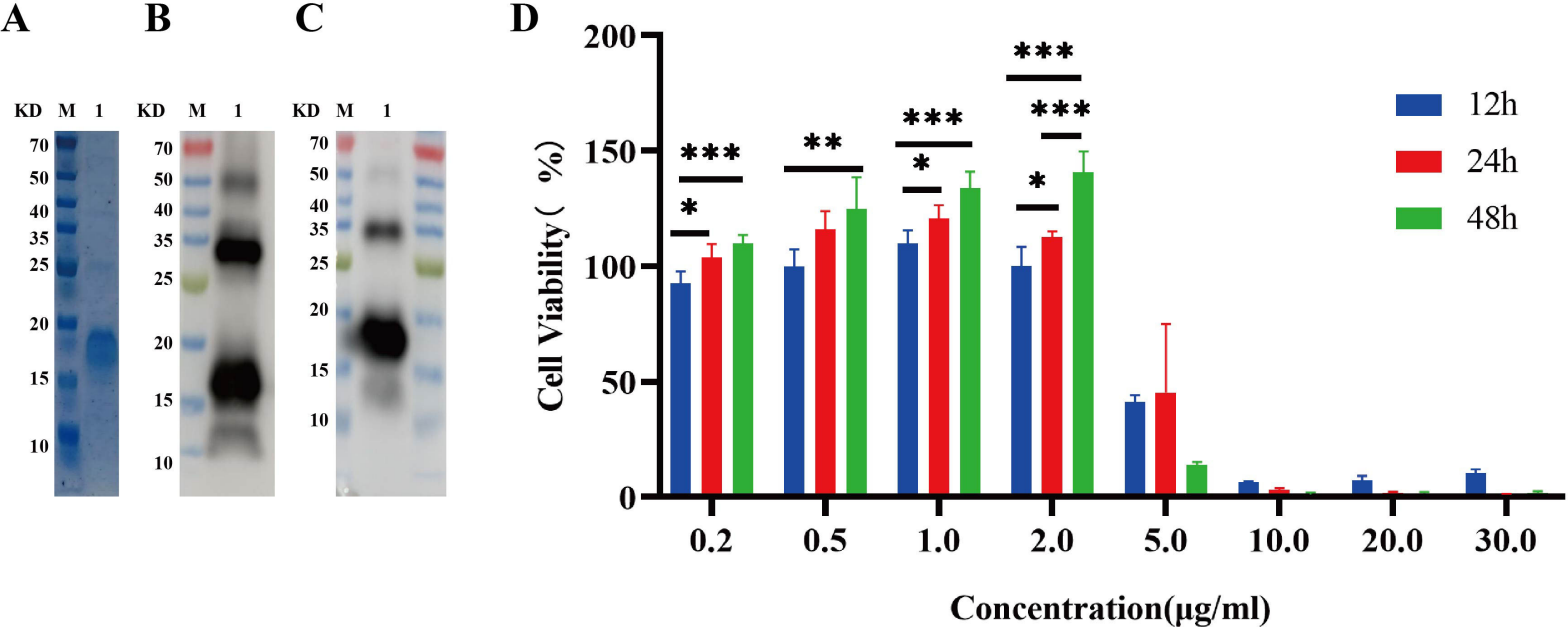
Expressions of recombinant IL-26 in *E. coli* and its effect on the viability of THP1 cells. **(A)** SDS-PAGE analysis of the purified recombinant IL-26. **(B, C)** Western blot analysis of the purified IL-26 using anti-His tag and anti-IL-26 monoclonal antibody, respectively. **(D)** The impact of varying concentrations of IL-26 on the viability of PMA-differentiated THP1 macrophages. *p < 0.05; **p < 0.01; ***p < 0.001 (one-way ANOVA with Tukey’s post-test).

Recombinant IL-26 was effectively overexpressed in the E. coli BL21 (DE3) strain at 37°C with the induction of 0.4 mM IPTG, as validated through comparison with the cells transformed with empty expression plasmid. Subsequently, the recombinant IL-26 was purified using the Ni-NTA resin. The SDS-PAGE analysis demonstrated high purity of the purified recombinant IL-26, with a molecular weight of 18 KD (**Figure 2A**). The purified recombinant IL-26 was further verified by western blot using the anti-His tag and anti-IL-26 monoclonal antibody, respectively (**Figures 2B, C**). Two distinct bands, corresponding to approximately 18 KD and 35 KD, were detected in both anti-His and anti-IL-26 immunoblotting testings, suggesting the presence of a dimeric form of IL-26. Furthermore, the effect of varying concentrations of recombinant IL-26 on the viability of human macrophage THP1 was examined using the CCK-8 approach. The results demonstrated that recombinant IL-26 at concentrations lower than 2μg/ml did not elicit detectable detrimental effects on THP1 macrophage viabilities (**Figure 2D**). Nevertheless, cell viability was markedly decreased when IL-26 reached 5μg/ml at 12 and 24 hours post-treatment (**Figure 2D**).

### IL-26 induces THP1 toward M1 polarization

**Figure 3:**
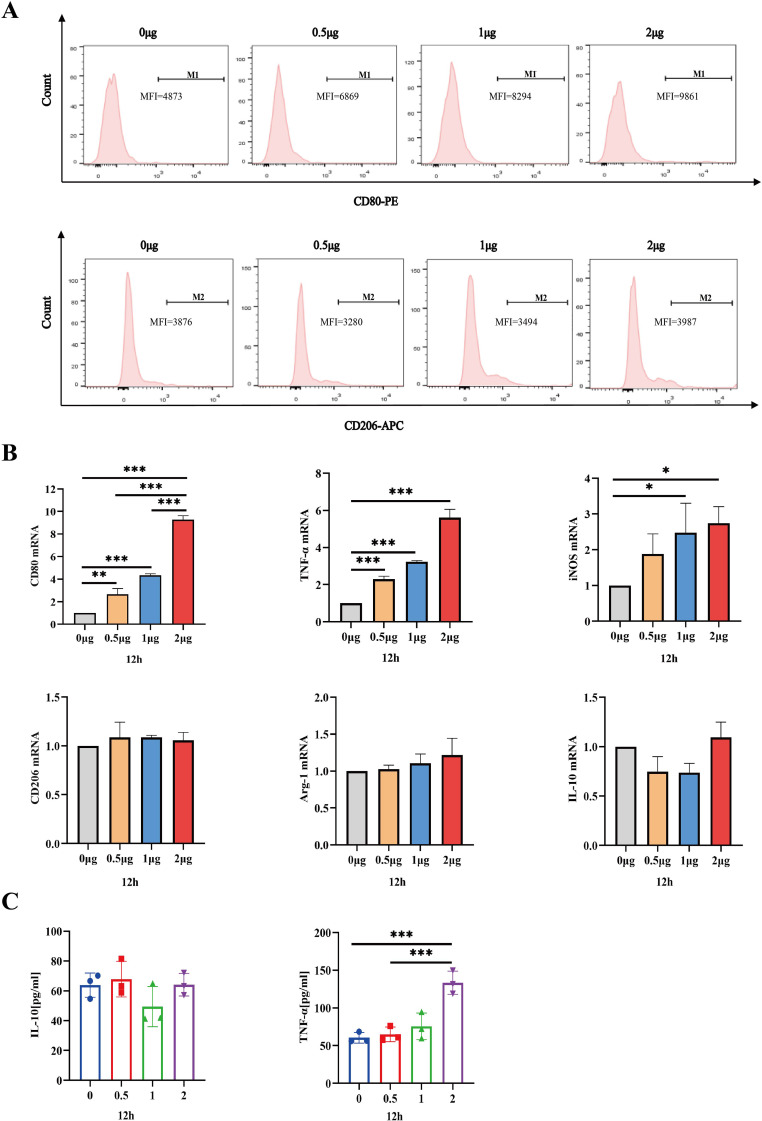
IL-26 Induces THP1 Toward M1 Polarization. **(A)** Flow cytometry analysis of CD80 and CD206 expressions in THP1 cells after exposure to varying concentrations of IL-26 for 24 hours. **(B)** The fold changes in mRNA expression of M1 macrophage marker genes (CD80, TNF-α, and iNOS) and M2 genes (CD206, IL-10, and Arg-1) in THP1 cells induced by varying concentrations of IL-26 for 12 hours were assessed by RT-PCR method. **(C)** Detections of secreted TNF-α and IL-10 in the supernatant of THP1 cells after treatment with varying concentrations of IL-26 for 12 hours by ELISA. All assays were performed in triplicate and were repeated at least three times. *p < 0.05; **p < 0.01; ***p < 0.001 (one-way ANOVA with Tukey’s post-test).

Having established that the safe dosage of IL-26 in THP1 cells was below 2μg/ml, we proceeded to investigate the impact of IL-26 on THP1 macrophage differentiation and polarization at doses of 0 μg/ml, 0.5 μg/ml, 1μg/ml, and 2μg/ml, respectively. Morphological alternations in THP1 macrophages were examined and imaged using phase contrast microscopy at 6, 12, and 24 hours post-treatment with IL-26. Results showed that cell populations with apparent spindle shape and abundant intracellular granules significantly increased in a dose- and time-dependent manner, indicating a shift of M0 macrophages toward the M1 phenotype after IL-26 treatment (**Supplementary Figure S1**). To further corroborate this IL-26-induced M1 polarization in THP1 cells, the expressions of surface marker CD80 for M1 macrophages and CD206 for M2 macrophages were assessed using the flow cytometry following stimulation with varying concentrations of IL-26. The findings revealed a dose-dependent significant increase of CD80 expressions on the surface of THP1 macrophages after 24 hours of exposure to different concentrations of IL-26 (**Figure 3A**). Conversely, no significant upregulation of CD206 expression was observed in THP1 cells treated with IL-26 compared to that of the control group cells (**Figure 3A**).

Moreover, the mRNA expression levels of M1 macrophage markers (CD80, TNF-α, and iNOS) and M2 markers (CD206, IL-10, and Arg-1) in THP1 cells stimulated with IL-26 for 24 hours were also assessed using the RT-PCR method. Similarly, significantly up-regulated mRNA expressions of CD80, TNF-α, and iNOS genes, indicative of M1 polarization, were observed in the THP1 cells treated with IL-26 for 6, 12, and 24 hours compared to the control group (**Figure 3B**; **Supplementary Figures S2A, C**). In contrast, there was no substantial change in CD206, IL-10, and Arg-1 gene expressions in THP1 cells after treatment with IL-26(**Figure 3B**; **Supplementary Figures S2B, D**). Moreover, the ELISA testing result also demonstrated a significant elevation of TNF-α secretion in THP1 cells stimulated with 2 μg/ml of IL-26 for 6, 12, and 24 hours compared to that of the control cells (**Figure 3C**; **Supplementary Figures S2E, F**). Increased IL-10 secretion induced by IL-26 in THP1 cells was only observed at 1 and 2 μg/ml concentrations for 24 hours instead (**Figure S2F**).

### IL-26 induces ROS production and mitochondrial damage in macrophages

**Figure 4:**
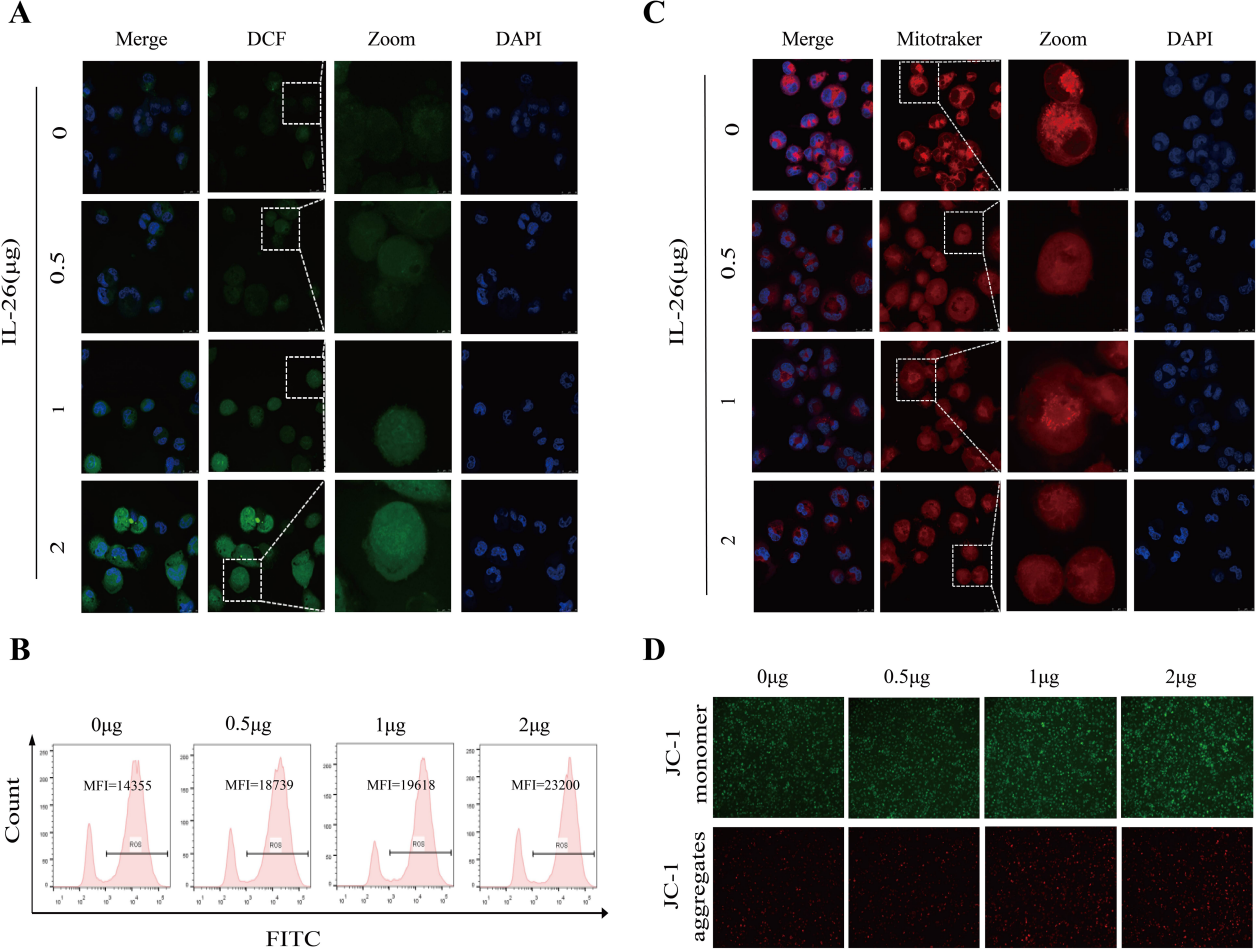
IL-26 Induces ROS Production and Mitochondrial Damage in Macrophages. **(A)** After treating the THP1 cells with varying concentrations of IL-26 for 24 hours, the cytoplasmic reactive oxygen species in THP1 cells were stained by the DCFH-DA probe and then visualized by confocal microscopy analysis. **(B)** Meanwhile, after the DCFH-DA probe staining, the mean fluorescence intensity (MFI) of these IL-26-stimulated THP1 was also measured using flow cytometry. **(C)** Confocal microscopy analysis of the impact of varying concentrations of IL-26 on the mitochondrial activity by Mitotracker staining. **(D)** The effect of different concentrations of IL-26 on mitochondrial membrane potential was examined using the JC-1 probe staining. All experiments were performed in triplicate and repeated at least three times.

M1 macrophages, also known as classically activated macrophages, are characterized by their high bactericidal activity and the production of pro-inflammatory cytokines and reactive oxygen and nitrogen species. Consequently, the effect of IL-26 on cytoplasmic reactive oxygen species production in THP1 macrophages was investigated utilizing the DCFH-DA probe staining approach. Cell fluorescence was assessed and quantified using confocal microscopy, a fluorescence plate reader, and flow cytometry analysis. All these analyses demonstrated a dose-dependent increase in green fluorescence intensity following IL-26 treatments for 24 hours, indicating that IL-26 can induce significant cytoplasmic ROS production in THP1 macrophages (**Figures 4A, B**; **Supplementary Figure S3A**).

Excessive ROS production typically leads to mitochondrial damage and dysfunction. Therefore, the impact of IL-26 on mitochondrial activity and mitochondrial ROS production was also examined using Mitotracker and MitoSOX probes staining, respectively. Utilizing confocal microscopy, we observed a decreased number and rupture of mitochondrial crista in IL-26-treated macrophages after staining by Mitotracker in a dose-dependent manner compared to the control group cells, indicating damaged mitochondria activity in IL-26-treated cells (**Figure 4C**). Furthermore, a significant decrease in red fluorescence intensity after Mitotracker staining in IL-26-treated macrophages was also recorded by fluorescence plate reader compared to the control group cells (**Supplementary Figure S3B**). For mitochondrial ROS production assays, our results revealed a significantly enhanced intensity of red fluorescence in IL-26-treated macrophages compared to that of the control group cells after MitoSOX staining, which demonstrated that IL-26 treatment significantly induced mitochondrial ROS production in THP1 macrophages (**Supplementary Figure S3C**).

On the other side, we also investigated the effect of IL-26 on the mitochondrial membrane potential in THP1 macrophages using the JC-1 probe staining. We found that compared to the control group cells, the IL-26-treated macrophages exhibited a significant increase of depolarized mitochondria, characterized by enhanced green fluorescence and decreased red fluorescence phenotype, as detected by both fluorescence microscopy and fluorescence microplate reader analysis (**Figure 4D**; **Supplementary Figure S3D**). Damaged mitochondria are generally degraded by a dedicated mitochondrial autophagy process, namely mitophagy, which plays a crucial role in maintaining cellular homeostasis. Our confocal microscopy analysis also confirmed a co-location of LC3 and mitochondria in IL-26-treated macrophages (**Supplementary Figure S4**). However, no significant difference in p-parkin/parkin expression, the mitophagy-associate markers, was observed in IL-26 treated macrophages compared to control cells (data not shown).

### IL-26 triggers ROS-mediated autophagy in macrophages

**Figure 5:**
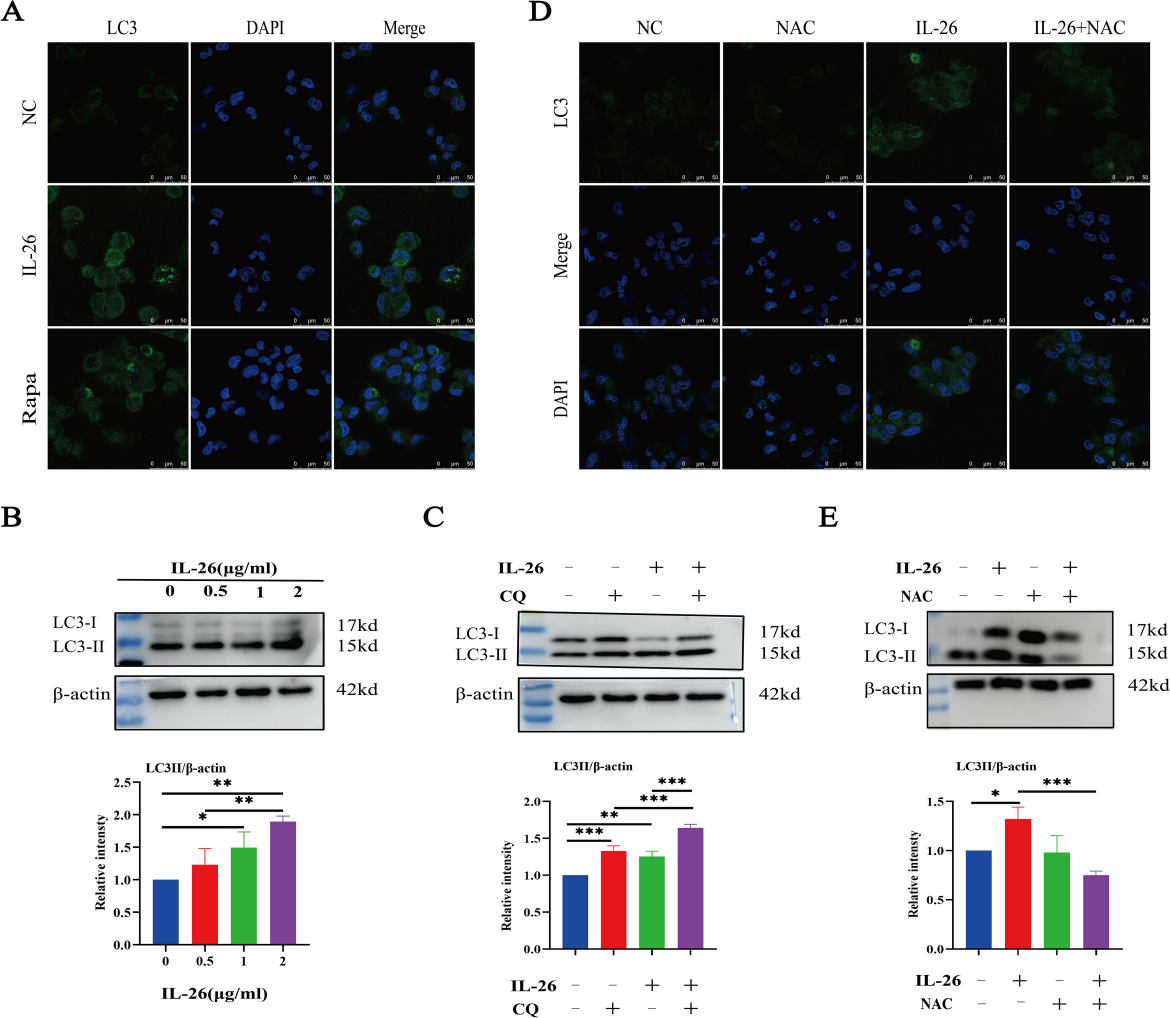
IL-26 Triggers ROS-mediated Autophagy in THP1 Macrophages. **(A)** Representative confocal microscopy images of LC3 protein staining in PMA-differentiated THP1 macrophages following treatment with either 2 µg/ml IL-26 or 300 nM rapamycin. **(B)** Western blot analysis of LC3 I/II protein level in THP1 cells with varying concentrations of IL-26. **(C)** Western blot analysis of LC3 I/II protein level in THP1 cells treated with 2 µg/ml IL-26, 25 µM CQ, or their combination. **(D, E)** Confocal microscopy and western blot analysis of LC3 protein level in PMA-differentiated THP1 cells treated with 2 µg/ml IL-26, 20 mM NAC, or their combination. Western blot images are a representative of at least three independent experiments. The quantitative analysis of band intensity from these experiments is presented below for each respective image. *p < 0.05; **p < 0.01; ***p < 0.001 (one-way ANOVA with Tukey’s post-test).

As we observed damaged mitochondria co-located with the autophagy protein LC3 in macrophages after IL-26 treatment, we hypothesize that the IL-26 treatment may be capable of inducing significant autophagy flux in THP1 macrophages. The autophagy-related protein LC3 puncta level and LC3b protein level were assessed using fluorescence microscopy and western blot analysis, respectively. Our results demonstrated that IL-26 treatment induced a significant increase in green LC3 puncta in THP1 macrophages, comparable to the level induced by rapamycin treatment (**Figure 5A**). In contrast, no apparent green LC3 puncta was detected in the control group cells. Moreover, western blot analysis also revealed an increased presence of LC3b in IL-26-treated THP1 macrophages compared to the control group cells, indicating that IL-26 induced the increased autophagy flux activity in macrophages (**Figure 5B**). In our subsequent experiment, the combination treatments of IL-26 and chloroquine, a drug that inhibits the fusion of autophagosomes with lysosomes, triggered significantly enhanced accumulations of LC3b in THP1 macrophages compared to the cells treated with IL-26 alone, suggesting that the chloroquine can inhibit the effect of IL-26 on inducing autophagy process in macrophages (**Figure 5C**). To further elucidate the relationship between the induced ROS and autophagy by IL-26 in macrophages, we examined the LC3b intensity in THP1 cells treated with IL-26 alone and in cells treated with a combination of IL-26 and NAC, a commonly used ROS scavenger, using both fluorescence microscopy and western blot analysis. Our results revealed a reduction of LC3b intensity in THP1 macrophages when NAC was simultaneously added, indicating that the induced autophagy process by IL-26 was mediated by the triggered ROS in macrophages (**Figure 5D, E**).

### IL-26 contributes to the elimination of intracellular *Mycobacterium tuberculosis* via induced ROS

**Figure 6:**
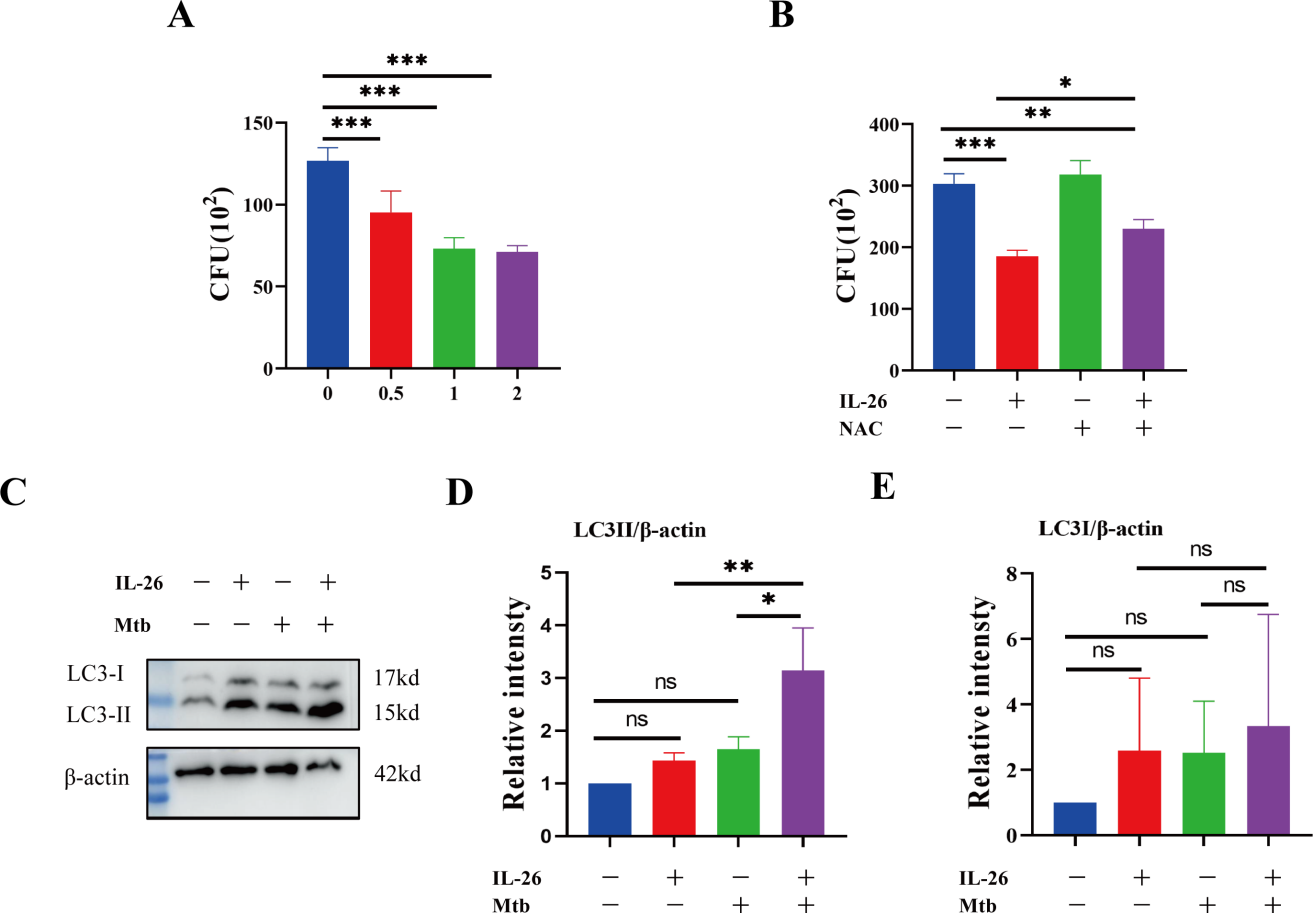
IL-26 contributes to the elimination of intracellular Mycobacterium tuberculosis via induced ROS. **(A)** The THP1 macrophages were infected with Mtb H37Ra at a multiplicity of 10 after being treated with varying concentrations of IL-26 for 24 hours. The cells were lysed, and the bacteria load was enumerated by plating at 24 Hours post-infections. **(B)** The THP1 were pre-treated with 2ug/ml IL-26, or 20 mM NAC or their combination, and then infected with Mtb H37Ra as the same multiplicity as above. Intracellular Mtb loads were enumerated by plating at 24 house post- infections as well. **(C)** The LC3 protein levels in THP1 cells that were infected with or without Mtb H37Ra or pre-treated with or without IL-26 were analyzed by western blotting. **(D, E)** The band intensity ratio between LC3II/I and the internal control β-actin from the above western blot analysis described in part **(C)** was quantified using image J. Assays were performed in triplicate and were repeated at least three times. *p < 0.05; **p < 0.01; ***p < 0.001 (one-way ANOVA with Tukey’s post-test).

Having established that IL-26 can induce THP1 toward M1 polarization characterized by enhanced ROS production, we further sought to determine whether IL-26 treatment could contribute to the intracellular elimination of *Mycobacterium tuberculosis* in THP1 macrophages. The PMA-differentiated THP1 macrophages were pre-treated with different concentrations of IL-26 for 24 hours and subsequently infected with Mycobacterium tuberculosis H37Ra at a multiplicity of 10:1 for six hours. At 24 hours post-infection, the cells were lysed, and the bacterial load was enumerated by plating. The results demonstrated a significantly lower intracellular Mtb H37Ra load in IL-26 pre-treated THP1 macrophages compared to non-pretreated cells, suggesting that IL-26 treatment enhances the intracellular killing of Mtb by macrophages (**Figure 6A**). To further elucidate whether the increased intracellular Mtb killing induced by IL-26 was associated with its triggered ROS production, we then examined the intracellular survival of Mtb in THP1 macrophages pre-treated with IL-26, NAC, or their combination. Notably, the intracellular Mtb load in cells treated with the combination of IL-26 and NAC was significantly lower than that in non-treated cells and cells treated with NAC alone but significantly higher than that in cells solely treated with IL-26 (**Figure 6B**). This finding indicated that the IL-26-induced intracellular Mtb killing is dependent on its triggered ROS production in THP1 macrophages. Furthermore, we also observed a significantly higher presence of LC3-II in THP1 macrophages that were pre-treated with IL-26 and subsequently infected with Mtb H37Ra, compared to cells only infected with Mtb H37Ra or cells solely treated with IL-26 (**Figure 6C–E**). This result suggests that IL-26 pretreatment can also enhance the autophagy flux during Mycobacterium tuberculosis infections.

## Discussions

IL-26 is an emerging pro-inflammatory member of the IL-10 family cytokines, which also includes IL-10, IL-19, IL-22, and IL-24. Recent research has reported significant elevations of IL-26 in individuals with different inflammatory conditions, including rheumatoid arthritis, Behçet’s disease, atopic dermatitis, psoriasis, asthma, and inflammatory bowel disease, indicating its multifaceted roles in antiviral, antimicrobial, and autoimmune responses (Meller et al., 2015; Stephen-Victor et al., 2016). In this study, we demonstrated that the IL-26 mRNA expression in PBMCs from active tuberculosis patients was significantly higher than that of healthy individuals. Interestingly, the levels of circulating IL-26 in the plasma of active tuberculosis patients were lower than those of healthy persons. We also demonstrated that IL-26 could induce the THP1 macrophages toward M1 polarization and contribute to the elimination of intracellular *Mycobacterium tuberculosis* via induced ROS-mediated response.

It is well-established that IL-26 is primarily produced and co-expressed with IL-22 by Th1, Th17, and NK cells (Donnelly et al., 2010). A previous study has documented a significant upregulation of IL26 mRNA expressions in tuberculous lymph nodes and cutaneous sarcoidosis tissues (Hawerkamp et al., 2020). Consistent with these findings, our research also observed a substantial increase in the IL26 mRNA expression in PBMC cells in tuberculosis patients. In contrast, the levels of circulating IL-26 in adult tuberculosis patients’ peripheral blood serum were found to be significantly lower than those in healthy individuals. A prior study conducted by Guerra-Laso et al. also showed a similar reduction in IL-26 levels in the serum of adult tuberculosis patients compared to healthy controls, although their study included only six tuberculosis patients and five healthy individuals. Based on these findings, we further confirmed that the serum levels of IL-26 in adult tuberculosis patients are lower than those in healthy individuals. Notably, a marked discrepancy exists between the up-regulated expression of IL-26 mRNA in PBMCs and the decreased circulating IL-26 levels in the plasma of tuberculosis patients. We hypothesize that several factors may contribute to this discrepancy. One possibility is the existence of multiple alternative splicing variants of IL-26 mRNA or post-transcriptional regulation of IL-26. Another possible reason is that the secreted IL-26 may be rapidly depleted or transported into infection sites during the blood circulation process in these adult tuberculosis patients as a response to combatting the disease. Indeed, our examination confirmed the abundant presence of IL-26 in the large granuloma of lung tissue in tuberculosis patients. We also analyzed some sample sections from patients with other lung diseases. After examining the relatively healthy areas of those lung tissues, we did not observe a significant presence of IL-26 except in regions of blood vessels(data not shown). These potential mechanisms warrant further investigations. Additionally, exploring the dynamics of IL-26 mRNA and protein levels before and after treatments in tuberculosis patients could also yield valuable insights into the role of IL-26 in tuberculosis pathogenesis.

Monocytes, macrophages and dendritic cells have been reported to respond to IL-26 with enhanced TNF-α secretion and increased expression of the chemokines CCL20, CXCL2, and CXCL8 (Hawerkamp et al., 2020). In addition, anti-CD40-stimulated B cells can respond to IL-26 with decreased secretion of immunoglobulin IgA and IgG (Hummelshoj et al., 2006; Tengvall et al., 2016). In our studies, we found that IL-26 treatment can trigger THP1 macrophages to up-regulate CD80, iNOS, and TNF-α expressions, which are gene markers of M1-type macrophages, in a dose-dependent manner, and this effect was not observed for marker genes of M2-type macrophages, such as CD206 and Arg-1. This suggests that IL-26 exposure can induce THP1 cells to differentiate into M1-type macrophages, which was also supported by morphological changes characterized by apparent spindle shape and abundant intracellular granules formed after IL-26 exposure. Interestingly, during initial examinations of the cytotoxic effect of different concentrations of recombinant IL-26 on THP1 macrophages, we demonstrated that doses less than 2 µg/ml have no noticeable detrimental effects on THP1 macrophage viabilities. However, approximately half of the THP1 cells were non-viable when the IL-26 dose reached 5 µg/ml. This surge in cell death was probably due to the excessive activation of THP1 macrophages, such as extremely high ROS production and mitochondrial damage induced by higher concentrations of IL-26. Similar significantly decreased cell viability and enhanced ROS productions have also been observed in different cell lines after treatment with increased concentrations of INF-γ (Rakshit et al., 2014).

Due to possessing the unique structure of six highly cationic α-helices, a hallmark of naturally occurring antimicrobial peptides, IL-26 is the only known Th17 cytokine capable of killing extracellular bacteria like *Staphylococcus aureus*, *Klebsiella pneumonia*, and *Escherichia coli* and so forth, via binding to the bacterial cell wall and causing pore formations and membrane disruption (Dang et al., 2019; Meller et al., 2015; Stephen-Victor et al., 2016). Recently, it was further recognized that IL-26 could exert its antimicrobial activity toward intracellular *Mycobacterium leprae* through two mechanisms (Dang et al., 2019). Firstly, IL-26 demonstrated direct antimicrobial activity against M. leprae, as evidenced by *in vitro* axenic culture and colonization with M. leprae in infected MDMs (Dang et al., 2019). Secondly, IL-26 was also able to induce the autophagy process for intracellular *M. leprae* clearance dependent on the cytoplasmic DNA receptor STING, in which the IL-26 likely binds to DNA from dying cells and then traffics this DNA to activate STING signaling for inducing autophagy (Dang et al., 2019; Meller et al., 2015; Poli et al., 2017; Watson et al., 2012). Our study also demonstrated that IL-26 could trigger the autophagy process in THP1 Macrophages, which is consistent with previous studies. However, our findings revealed that the induced autophagy by IL-26 in THP1 macrophages was also mediated by its induced ROS because the presence of the ROS scavenger NAC could significantly inhibit the autophagy flux and the antimicrobial activity of IL-26 in the context of *Mycobacterium tuberculosis* infections. Despite the absence of IL-26 in murine models, future research holds promise for investigating the protective role of IL-26 in the elimination of Mycobacterium tuberculosis infections in non-human primate models.

In conclusion, our study revealed significant differences in IL-26 expression in PBMC and circulating IL-26 levels in the serum of tuberculosis patients compared to healthy individuals. Moreover, our findings demonstrated that IL-26 treatment could drive THP1 cells toward M1-type macrophage polarization and induce ROS production and autophagy processes in these macrophages. These effects collectively contribute to the elimination of intracellular *Mycobacterium tuberculosis*, which plays a crucial role in the host defense against Mtb infection.

## Data Availability

The raw data supporting the conclusions of this article will be made available by the authors, without undue reservation.

## Supplementary material

Supplementary Figure 1 Morphological changes of THP1 cells after exposure to IL-26 treatment. Cell populations with apparent spindle shape and abundant intracellular granules significantly increased upon IL-26 treatment, suggesting a shift from M0 macrophages toward the M1 phenotype.

Supplementary Figure 2 IL-26 promoted THP1 Toward M1 Polarization. **(A, B)** The fold changes in mRNA expression levels of M1 marker genes (CD80, TNF-α, and iNOS) and M2 marker genes (CD206, IL10, and Arg-1) in THP1 cells at 6 hours post IL-26 exposure, as quantified using the RT-PCR method. **(C, D)** The fold changes in mRNA expression levels of M1 marker genes (CD80, TNF-α, and iNOS) and M2 marker genes (CD206, IL10, and Arg-1) in THP1 cells at 24 hours post IL-26 exposure measured by the RT-PCR method. **(E, F)** Quantifications of TNF-α and IL-10 secretions were performed at 6 and 24 hours post IL-26 exposure by the ELISA method. All experiments were performed in triplicate and repeated at least three times.

Supplementary Figure 3 ROS productions and Mitochondrial damage induced by IL-26. **(A, C)** The cytoplasmic ROS and mitochondrial ROS production in THP1 macrophage after treatment with varying concentrations of IL-26 for 24 hours was assessed using the DCFH-DA and MitoSOX Red probe. The fluorescence intensity was measured using a fluorescent microplate reader. **(B, D)** Mitochondrial activity and membrane potential in THP1 cells after IL-26 exposure were evaluated using the MitoTracker Red probe and JC-1 probe staining, and the fluorescence intensity was measured employing a fluorescent microplate reader.

Supplementary Figure 4 Co-localization of LC3 proteins with the mitochondrial in THP1 macrophages following treatment with 2 µg/ml IL-26 for 24 hours.
