# Supplementary material for: Interleukin-26 expression in tuberculosis disease and its regulatory effect in macrophage polarization and intracellular elimination of Mycobacterium tuberculosis
Source: Front Cell Infect Microbiol. 2024 Oct 4;14:1455819. doi: 10.3389/fcimb.2024.1455819 (PMC11486762; doi:10.3389/fcimb.2024.1455819)
Supplement: Supplementary file 2 [file Table1.docx]

Supplementary Table 1

| RT-PCR Gene | Primer sequence |
| --- | --- |
| GAPDH | F:5'-GGTCTCCTCTGACTTCAACA-3' |
|  | R:5'-GTGAGGGTCTCTCTCTTCCT-3' |
| IL-26 | F:5'-AGCAACGATTCCAGAAGACC-3' |
|  | R:5'-TGAAAGTCCTCCACAAAGCGTA-3' |
| CD80 | F:5'-GGGAAATGTCGCCTCTCTGAA-3' |
|  | R:5'-CCTGGGTCTCCAAAGGTTGT-3' |
| TNF-α | F:5'-GTGACAAGCCTGTAGCCCATGTT-3' |
|  | R:5'-TTATCTCAGCTCCACGCCATTA-3' |
| iNOS | F:5'-TTCAGTATCACAACCTCAGCAAG-3' |
|  | R:5'-TGGACCTGCAAGTTAAAATCCC-3' |
| CD206 | F:5'-ACCTGCGACAGTAAACGAGG-3' |
|  | R:5'-TGTCTCCGCTTCATGCCATT-3' |
| Arg-1 | F:5'-TTCTCAAAGGGACAGCCACG-3' |
|  | R:5'-CGCTTGCTTTTCCCACAGAC-3' |
| IL-10 | F:5'-TGAAGAATGCCTTTAATAAGCTCCA-3' |
|  | R:5'-ATAGAGTCGCCACCCTGATG-3' |
| F, forward; R, reverse | |
