# Supplementary figures and images for: Interleukin-26 expression in tuberculosis disease and its regulatory effect in macrophage polarization and intracellular elimination of Mycobacterium tuberculosis
Source: Front Cell Infect Microbiol. 2024 Oct 4;14:1455819. doi: 10.3389/fcimb.2024.1455819 (PMC11486762; doi:10.3389/fcimb.2024.1455819)

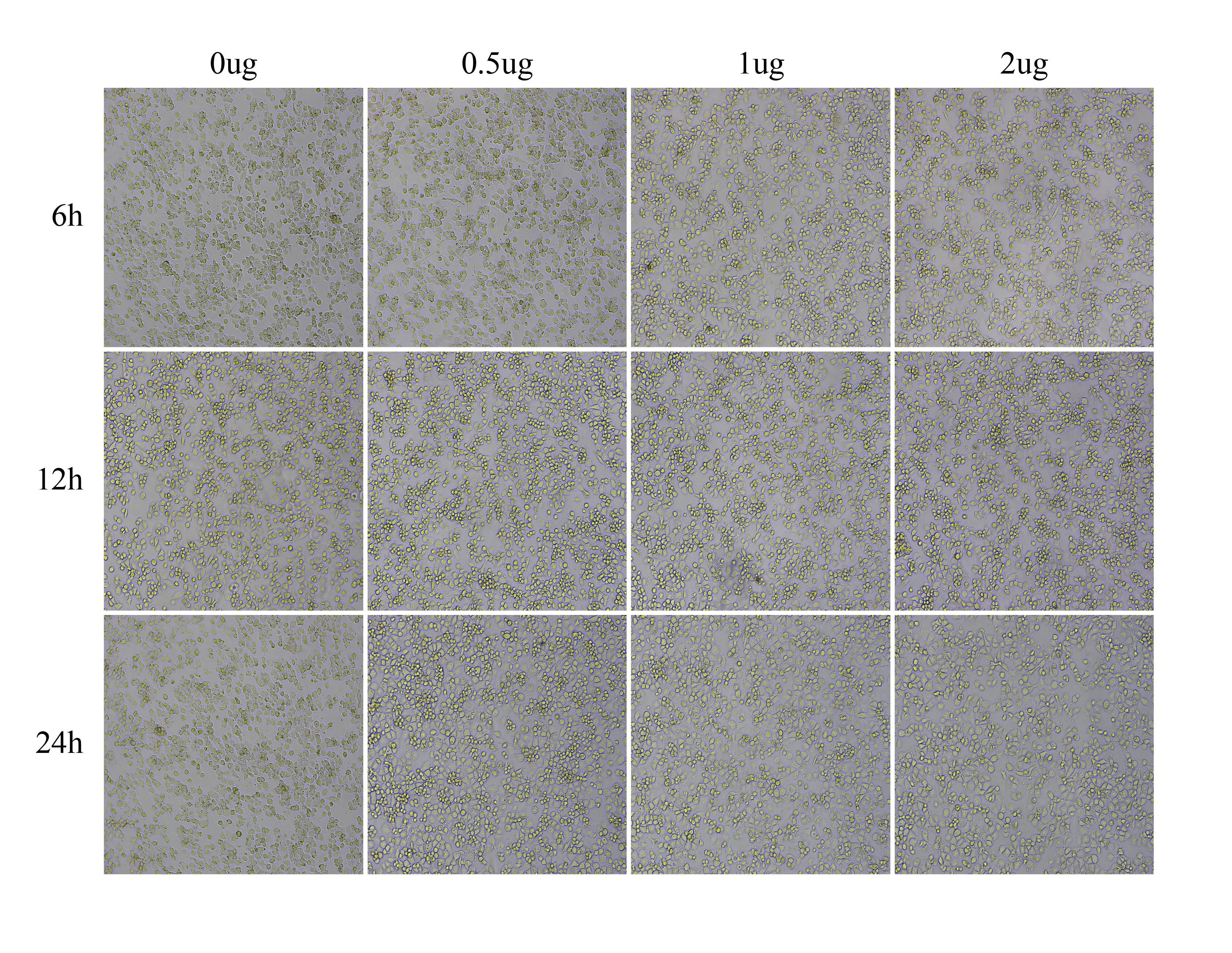

Supplement: Supplementary Figure 1 — Morphological changes of THP1 cells after exposure to IL-26 treatment. Cell populations with apparent spindle shape and abundant intracellular granules significantly increased upon IL-26 treatment, suggesting a shift from M0 macrophages toward the M1 phenotype. [file Image1.jpeg]

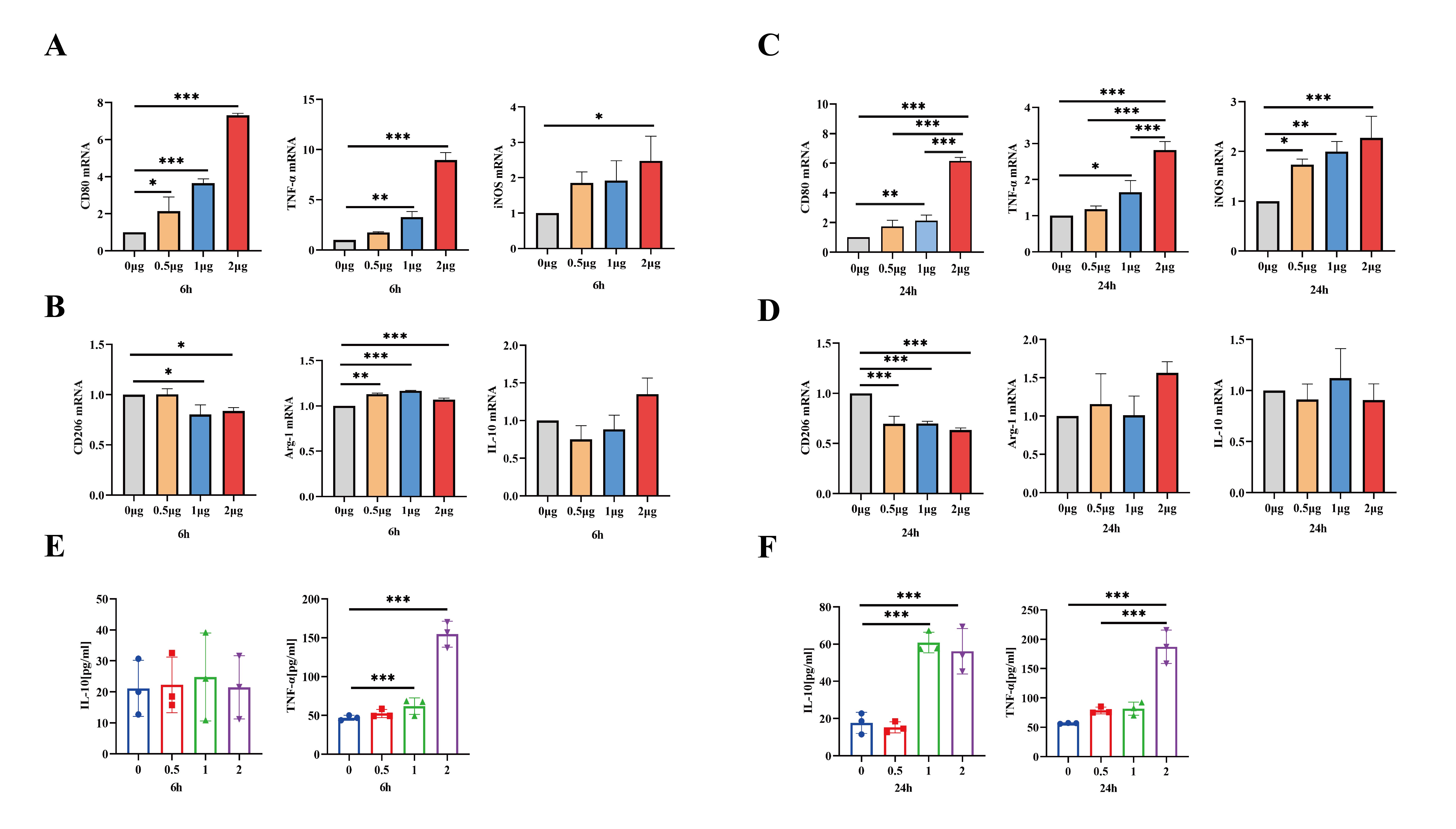

Supplement: Supplementary Figure 2 — IL-26 promoted THP1 Toward M1 Polarization. (A, B) The fold changes in mRNA expression levels of M1 marker genes (CD80, TNF-α, and iNOS) and M2 marker genes (CD206, IL10, and Arg-1) in THP1 cells at 6 hours post IL-26 exposure, as quantified using the RT-PCR method. (C, D) The fold changes in mRNA expression levels of M1 marker genes (CD80, TNF-α, and iNOS) and M2 marker genes (CD206, IL10, and Arg-1) in THP1 cells at 24 hours post IL-26 exposure measured by the RT-PCR method. (E, F) Quantifications of TNF-α and IL-10 secretions were performed at 6 and 24 hours post IL-26 exposure by the ELISA method. All experiments were performed in triplicate and repeated at least three times. [file Image2.jpeg]

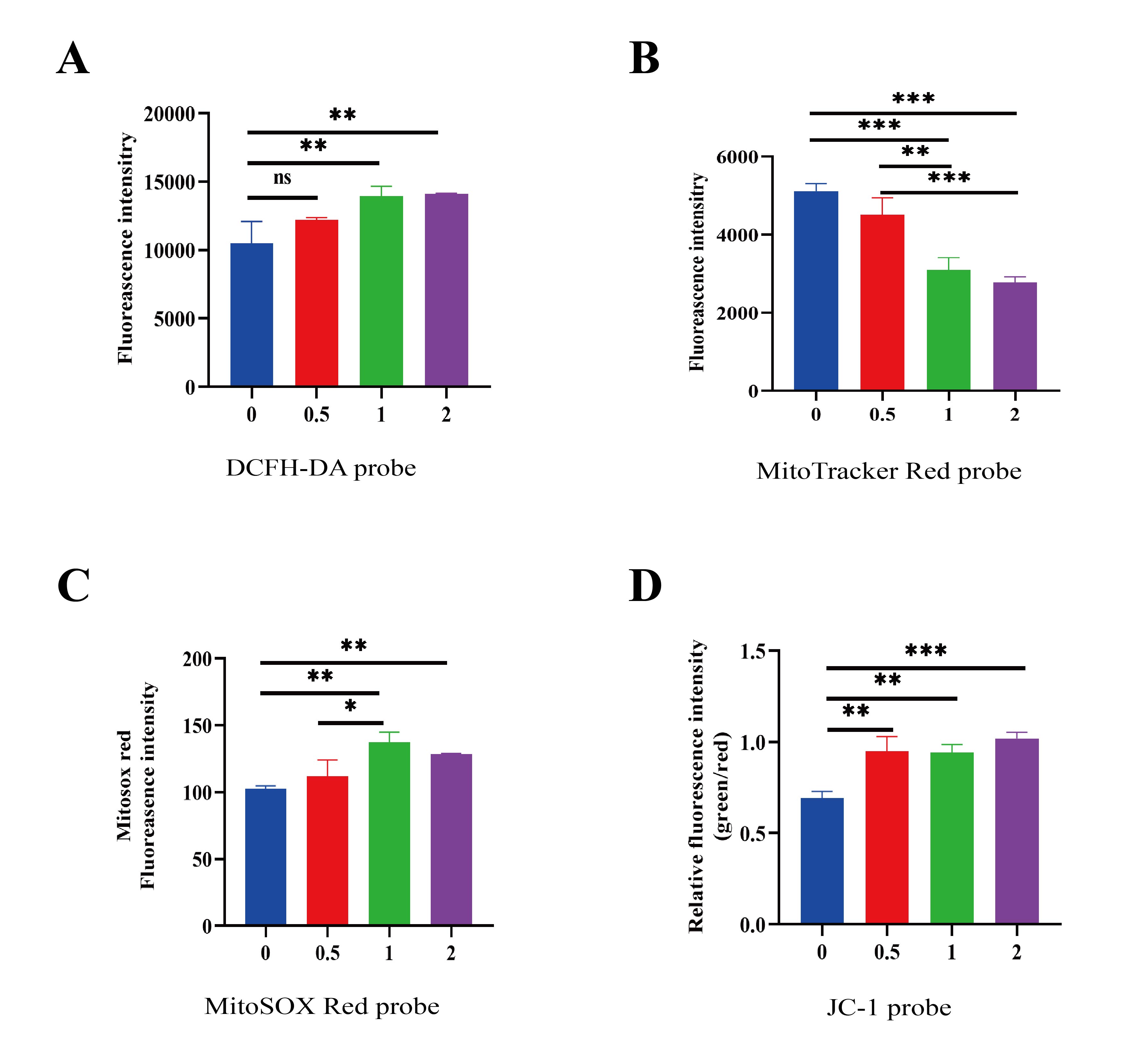

Supplement: Supplementary Figure 3 — ROS productions and Mitochondrial damage induced by IL-26. (A, C) The cytoplasmic ROS and mitochondrial ROS production in THP1 macrophage after treatment with varying concentrations of IL-26 for 24 hours was assessed using the DCFH-DA and MitoSOX Red probe. The fluorescence intensity was measured using a fluorescent microplate reader. (B, D) Mitochondrial activity and membrane potential in THP1 cells after IL-26 exposure were evaluated using the MitoTracker Red probe and JC-1 probe staining, and the fluorescence intensity was measured employing a fluorescent microplate reader. [file Image3.jpeg]

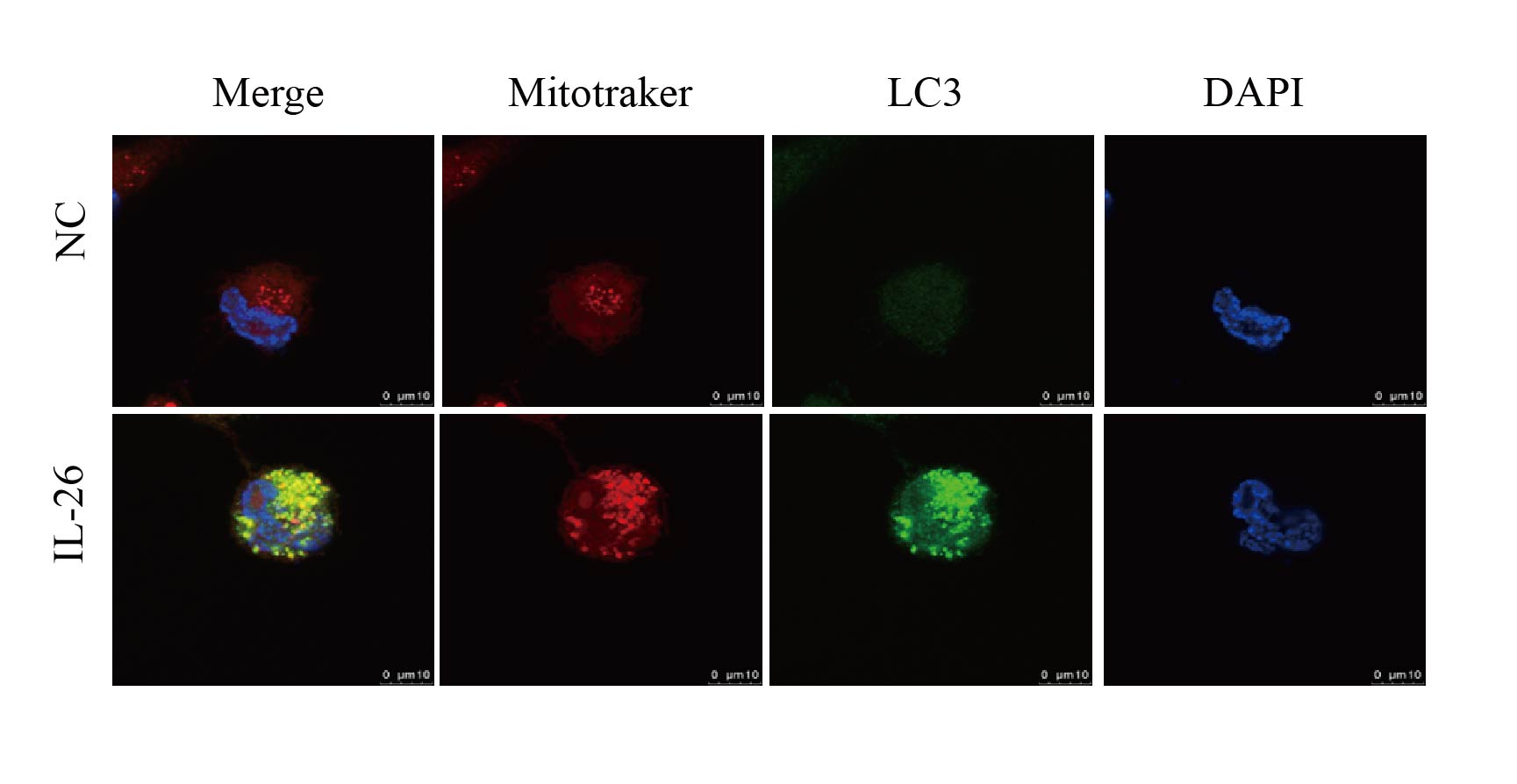

Supplement: Supplementary Figure 4 — Co-localization of LC3 proteins with the mitochondrial in THP1 macrophages following treatment with 2 µg/ml IL-26 for 24 hours. [file Image4.jpeg]
